# Infection of Apple by *Apple Stem Grooving Virus* Leads to Extensive Alterations in Gene Expression Patterns but No Disease Symptoms

**DOI:** 10.1371/journal.pone.0095239

**Published:** 2014-04-15

**Authors:** Shanyi Chen, Ting Ye, Lu Hao, Hui Chen, Shaojie Wang, Zaifeng Fan, Liyun Guo, Tao Zhou

**Affiliations:** State Key Laboratory of Agrobiotechnology and Department of Plant Pathology, China Agricultural University, Beijing, China; The Ohio State University, United States of America

## Abstract

To understand the molecular basis of viral diseases, transcriptome profiling has been widely used to correlate host gene expression change patterns with disease symptoms during viral infection in many plant hosts. We used infection of apple by *Apple stem grooving virus* (ASGV), which produces no disease symptoms, to assess the significance of host gene expression changes in disease development. We specifically asked the question of whether such asymptomatic infection is attributed to limited changes in host gene expression. Using RNA-seq, we identified a total of 184 up-regulated and 136 down-regulated genes in apple shoot cultures permanently infected by ASGV in comparison with virus-free shoot cultures. As in most plant hosts showing disease symptoms during viral infection, these differentially expressed genes encode known or putative proteins involved in cell cycle, cell wall biogenesis, response to biotic and abiotic stress, development and fruit ripening, phytohormone function, metabolism, signal transduction, transcription regulation, translation, transport, and photosynthesis. Thus, global host gene expression changes do not necessarily lead to virus disease symptoms. Our data suggest that the general approaches to correlate host gene expression changes under viral infection conditions to specific disease symptom, based on the interpretation of transcription profiling data and altered individual gene functions, may have limitations depending on particular experimental systems.

## Introduction

Plant viral infection causes enormous economic losses in many crops worldwide. To understand the molecular basis of virus diseases, many studies have recently investigated host global gene expression changes in plant-virus interactions [1]–[13]. However, these studies have focused on viral infection of herbaceous plants producing visible disease symptoms. The molecular interactions of viruses with woody species, including economically important fruit trees, are poorly studied. Thus far only one study analyzed transcriptome changes in a woody perennial plant in response to virus infection: grapevine (*Vitis vinifera* L.) infected by *Grapevine rupestris stem pitting-associated virus* [14]. In general, transcriptome analyses of virus-infected plants detected changes in the expression patterns of host genes encoding diverse functions. Although up- or down-regulated expressions of these genes were often discussed with respect to specific signaling networks or to their possible individual roles in viral infection and host response, it has been difficult to derive conclusive models for symptom expression.

Here, we used infection of apple by *Apple stem grooving virus* (ASGV) to gain further insights into molecular responses of a woody host species to viral infection and to assess the significance of host gene expression changes in disease development. Apple (*Malus*×*domestica* Borkh.) is one of the most widely grown fruit crops in the world. It is an important source of energy, vitamins and minerals in human diet. Apple is susceptible to infection by pathogens, especially viruses such as *Apple stem grooving virus* (ASGV), *Apple chlorotic leaf spot virus* (ACLSV) and *Apple stem pitting virus* (ASPV). These viruses usually do not induce visible disease symptoms in the infected trees and fruits, although the infection eventually does lead to significant reduction in fruit yield and quality [15]–[17]. Further, the infection is permanent in apple trees due to vegetative propagation [16].

ASGV is the type member of genus *Capillovirus* in the family *Betaflexiviridae* [18]. It has a single-stranded RNA genome of approximately 6500 nucleotides and virion particles of 600–700 nm in length. The genome has two overlapping open reading frames (ORFs) encoding proteins of 241 and 36 kDa, respectively [19]-[21]. ORF1 encodes a polyprotein containing a replication-associated protein plus a coat protein (CP), and ORF2 encodes a movement protein located within ORF1 in a different reading frame. CP is expressed from a subgenomic RNA [22] and is essential for infection [23].

The ASGV-infected *in vitro* apple shoots are asymptomatic, providing an excellent model system to assess the significance of transcriptome changes in disease symptom development. An outstanding question is whether such asymptomatic infection is attributed to limited changes in host gene expression patterns, in contrast to extensive gene expression changes observed in other plants exhibiting visible disease symptoms. To address this question, we used RNA-seq to analyze the transcriptome profile of *in vitro* apple shoots permanently infected by ASGV in comparison with that of virus-free *in vitro* shoots. Our analyses revealed extensive changes in the apple gene expression patterns under ASGV infection, despite absence of visible disease symptoms, similar to those reported for other plant-virus pathosystems in which the infected hosts showed clear symptoms. We present our findings and discuss their biological implications.

## Materials and Methods

### Elimination of ASGV by thermotherapy

*In vitro*-grown plantlets of apple cv. Fuji were cultured in a medium (pH 5.8) composed of Murashige and Skoog medium (MS) containing 3% sucrose, 1 mg/L 6-benzylaminopurine (6-BA) and 0.02 mg/L 1-naphthlcetic acid (NAA) solidified with 0.8% agar powder. Stock cultures were maintained at a temperature of 25±1°C under a 16-h light/8-h dark photoperiod with a light intensity of 3600 Lx by cool-white fluorescent tubes. Subculturing was performed every 4 weeks.

For virus elimination, well-developed single shoots (>2 cm in length) were excised from ASGV-infected stock cultures and cultured in 150 mL triangular flasks. After 7 days, the flasks were transferred to a heat chamber programmed for a cycle of 16-h light at 37°C followed by an 8-h darkness at 34°C for 38 days. After thermotherapy, meristems of 1 mm were excised under a microscope and cultured for 7 days in solid MS medium containing 3% sucrose and supplemented with 1 mg/L 6-BA, 0.2 mg/L NAA and 0.5 mg/L gibberellin A3 at a temperature of 25°C in the dark for growth and plant regeneration. Regenerated shoots were subjected to virus detection after 60 days. RT-PCR and Northern blotting were performed to detect ASGV, ACLSV, ASPV, *Apple skin scar viroid* (ASSVd) and *Apple dimple fruit viroid* (ADFVd) in the regenerated shoots with specific primers (Supplementary Table S1). The ASGV-infected shoots were observed for two years in 20 generations and no symptoms were observed.

### RNA isolation, different gene expression library preparation and sequencing

Regenerated shoots were individually divided into ASGV-infected plantlets and virus-free plantlets based on detection result of RT-PCR and Northern blotting, 60 days after culturing. Before RNA extraction for cDNA library construction, every plantlet was confirmed to be ASGV-infected or virus-free by RT-PCR and Northern blotting. For library construction, the two RNA pools were obtained, separately, from 10 ASGV-infected plantlets and 10 virus-free plantlets. Total RNA was extracted from mixed samples according to the method described by [24]. After treatment with DNase I and extraction with phenol/chloroform and precipitation, RNA pellets were dissolved in 30 µL of RNase-free water and visualized in a 1.0% agarose gel and quantified using a NanoDrop ND-2000 Spectrophotometer (NanoDrop Technologies, Wilmington, Delaware, USA).

The libraries for RNA-seq were prepared from equal amounts of total RNA from different samples. Following the manufacturer's instructions (Illumina, San Diego, California, USA), mRNA was first purified from 20 µg of total RNA using oligo(dT) magnetic beads and then fragmented into small pieces in the fragmentation buffer. The cleaved RNA fragments were used for reverse transcription followed by second-strand cDNA synthesis using DNA polymerase I and RNase H. The double-stranded cDNA was purified with QiaQuick PCR extraction kit (QIAGEN, Dusseldorf, Germany) and washed with elution buffer for end repair and single adenine addition. Finally, sequencing adaptors were ligated to the fragments. The required fragments were purified by agrose gel electrophoresis and enriched by PCR amplification. The library products were used for sequencing via the Illumina HiSeq 2000 system (Illumina, San Diego, California, USA).

After sampling the plantlets were continuously cultured and propagated under the same growth conditions.

### Tag annotation and data normalization for gene expression levels

Raw RNA sequences were cleaned by removal of adaptor sequences, N-containing reads with more than 10% of unknown bases, low-quality sequences containing 50% bases of quality value ≤5, and adaptor-alone sequences. The clean reads were mapped to reference sequences (Malus×domestica Whole Genome v1.0) in the Genome Database of Rosaceae (http://www.rosaceae.org/species/malus/malus_x_domestica/genome_v1.0) [25] using SOAPaligner/soap2 [26] allowing no more than 2 nucleotide mismatches. The reads mapped to multiple gene sequences were filtered, and the remaining reads were designated as unambiguous reads. For gene expression analysis, the number of unambiguous reads for each gene was calculated and then normalized to reads per kb per million reads (RPKM) [27]. The gene ontology (GO, http://www.geneontology.org/) classification system and Uniprot database (http://www.uniprot.org/) were used to infer the functions of all genes. RNA-Seq data have been deposited in NCBI's Gene Expression Omnibus under accession number GSE53825 (http://www.ncbi.nlm.nih.gov/geo/query/acc.cgi?acc=GSE53825).

### Analysis of differential gene expression

Fisher's exact test [28] was used to identify differentially expressed genes (DEG) between the virus-free and ASGV-infected plantlets. False discovery rate (FDR) was used to determine the threshold *P* value in multiple tests and analysis. We used an FDR of <0.001 and the absolute value of log_2_ ratio ≥1 as the threshold to judge the significance of gene expression differences [29].

### Quantitative real-time reverse-transcription PCR (qRT-PCR) validation

To validate the DEG results, qRT-PCR was performed with RNA samples prepared following the method for RNA-seq library construction. Ten ASGV-infected and 10 virus-free *in vitro* grown plantlets were pooled separately. Total RNA was extracted from each pool and subjected to DNase I treatment (TaKaRa, Dalian, China). The first-strand cDNA synthesis was performed with Oligo(dT) primer and random hexamer primer using M-MLV reverse transcriptase (Promega, Madison, Wisconsin, USA) with 2 µg of total RNA according to the manufacturer's instructions. Eight randomly selected genes with relevant expression profiles from the RNA-seq data were tested. Specific primers (Supplementary Table S1) were designed using DNAMAN (v5.2.2) software. The SYBR Green real-time PCR assay was carried out in a total volume of 20 µL that contained 10 µL of 2×SYBR Premix *Ex Taq* II (Tli RNaseH Plus) (TaKaRa, Dalian, China), 0.4 µM (each) specific primers, 0.4 µL ROX Reference Dye II (50×), and 100 ng of template cDNA. The amplification program consisted of one cycle of 95°C for 30 s followed by 40 cycles of 95°C for 5 s and 60°C for 34 s. The fluorescent product was detected in the last step of each cycle. Following the amplification, melting temperatures of PCR products were analyzed to determine the specificity of the PCR products. Melting curves were obtained by slow heating at 1.6°C/s, from 60°C to 95°C, while continuously monitoring the fluorescence signal. A negative control without a cDNA template was run with each analysis to evaluate the overall specificity. Amplifications were carried out in 8 strip tubes (0.2 mL) in a ViiA7 Real Time PCR System (Applied Biosystems). All samples were run in triplicate. Amplification of an apple *ACTIN* gene (MDP0000774288) was used as an internal control. In total, three biological replicates (with 10 plantlets pooled for each replicate) were used for qRT-PCR analyses to obtain an average value. The average values from the three biological replicate were then used to calculate the mean and standard error. Using the 2^-ΔΔCT^ method, the data of relative gene expression were analyzed [30].

### Northern blotting

Total RNA was separated on a 1.2% agarose gel containing formaldehyde and transferred to a Hybond-N^+^ membrane (GE Healthcare Life Sciences, Piscataway, New Jersey, USA) according to manufacturer's protocol. After baking at 80°C for 2 h, the membranes were hybridized with ^32^P-labeled DNA probes. Prehybridization (1 h) and hybridization were carried out at 68°C in a hybridization solution containing 0.4 M Na_2_HPO_4_ (pH 7.0), 7% SDS, 1% bovine serum albumin (BSA) and 0.02 M EDTA. The membranes were washed twice for 20 min with 2×SSC and 2% SDS and once for 20 min with 0.2×SSC and 0.2% SDS at 68°C. Typhoon Trio Variable Imager (GE Healthcare, Uppsala, Sweden) was used for signal detection and analysis.

### Measurement of photosynthetic parameters

Micropropagated apple plantlets were transferred to a rooting medium consisting of half-strength MS salts, 30 g/L sucrose, 8 g/L agar and 1 mg/L indole-3-butyric acid. The cultures were maintained at 25±2°C under a 16/8-h (light/dark) photoperiod with light supplied by cool-white daylight fluorescent light bulbs. After acclimatizing with the Hoagland solution, the rooted shoots were transferred to soil and grown in a greenhouse. The fourth fully expanded leaf from a plantlet was used to measure the main photosynthetic parameters, including net photosynthetic rate (Pn), stomatal conductance (g_s_), substomatal CO_2_ concentration (Ci) and transpiration rate (Tr), with a portable LI-6400 photosynthesis system (Li-Cor, Lincoln, Nebraska, USA) under a constant leaf temperature of 20°C and alterable illumination supplied by the LI 6400-02B light system (Li-Cor). An average value for each parameter was obtained for each leaf based on five independent measurements. A total of six leaves from six ASGV-infected and six leaves from six virus-free plantlets, respectively, were used for the measurement. The six averages were then used to calculate the mean and standard error for each parameter for ASGV-infected and for virus-free plantlets, respectively. The data were statistically analyzed by *t*-test with statistical analysis system (SAS) software [31].

## Results

### Production of virus-free *in vitro*-grown apple plantlets via meristem tip culture and thermotherapy

**Figure 1:**
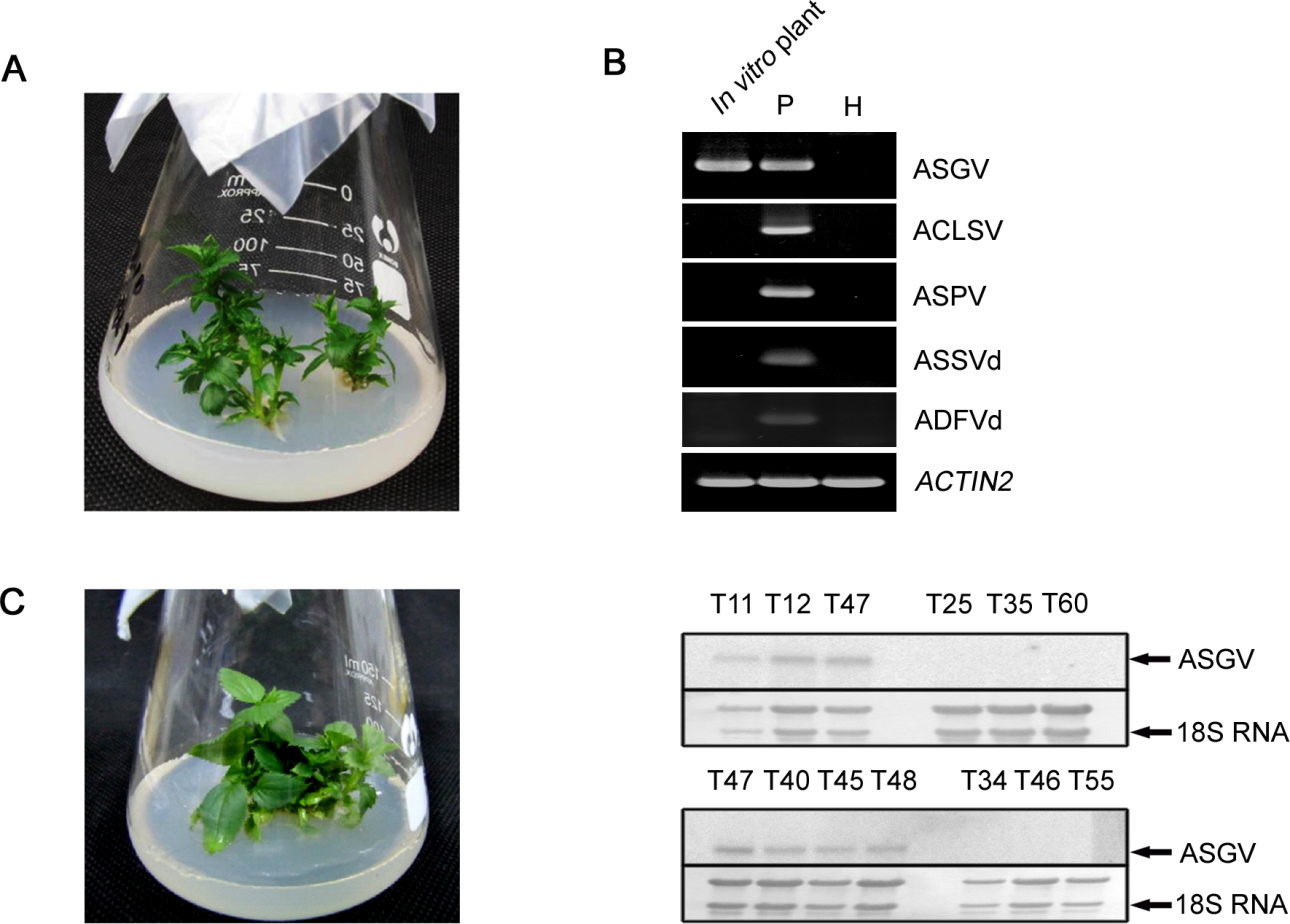
Virus-free and ASGV-infected plantlets for RNA-Seq. (A) ASGV-infected *in vitro* grown plantlets. (B) Detection of *Apple stem grooving virus* (ASGV), *Apple chlorotic leaf spot virus* (ACLSV), *Apple stem pitting virus* (ASPV), *Apple skin scar viroid* (ASSVd) and *Apple dimple fruit viroid* (ADFVd) using RT-PCR with specific primers listed in Supplementary Table S1. *ACTIN2* gene was used as an internal control. P, positive apple samples infected with ASGV, ACLSV, ASPV, ASSVd and ADFVd; H, healthy apple leaves. (C) Virus-free plantlets and Northern blotting to confirm ASGV elimination by thermotherapy. T11, T12, T25, T34, T35, T40, T45, T46, T47, T48, T55 and T60 represent regenerated apple plantlets after thermotherapy.

*In vitro*-grown apple shoots, derived by vegetative propagation from a single mother plant, were propagated in growth chambers (Fig. 1A). RT-PCR using specific primers (Supplementary Table S1) showed that there was only ASGV in the cultured *in vitro* plantlets (Fig. 1B). The genome sequence of the ASGV isolate (KF434636 in GenBank) had the highest identity to that of the first isolate (NC_0011749) reported from Japan [19], not only for the whole genome sequence (97.6%) but also for ORF1, ORF2, and the CP at the nucleotide (97.2%–98.5%) and amino acid (96.9–99.6%) levels (H. Chen and T. Zhou, unpublished data). To obtain virus-free micropropagated plantlets, a total of 150 shoots were subjected to thermotherapy, and 62 shoot tips were excised and cultured for regeneration. Among them, 58 developed into plantlets. RT-PCR showed that six regenerated plantlets were virus-free (data not shown). This was confirmed by Northern blotting with an ASGV CP-specific probe (Fig. 1C). Multiple propagations of regenerated ASGV-infected and virus-free *in vitro* plantlets were performed to obtain enough samples for gene expression profiling.

### RNA-seq and tag mapping to the apple genome

**Figure 2:**
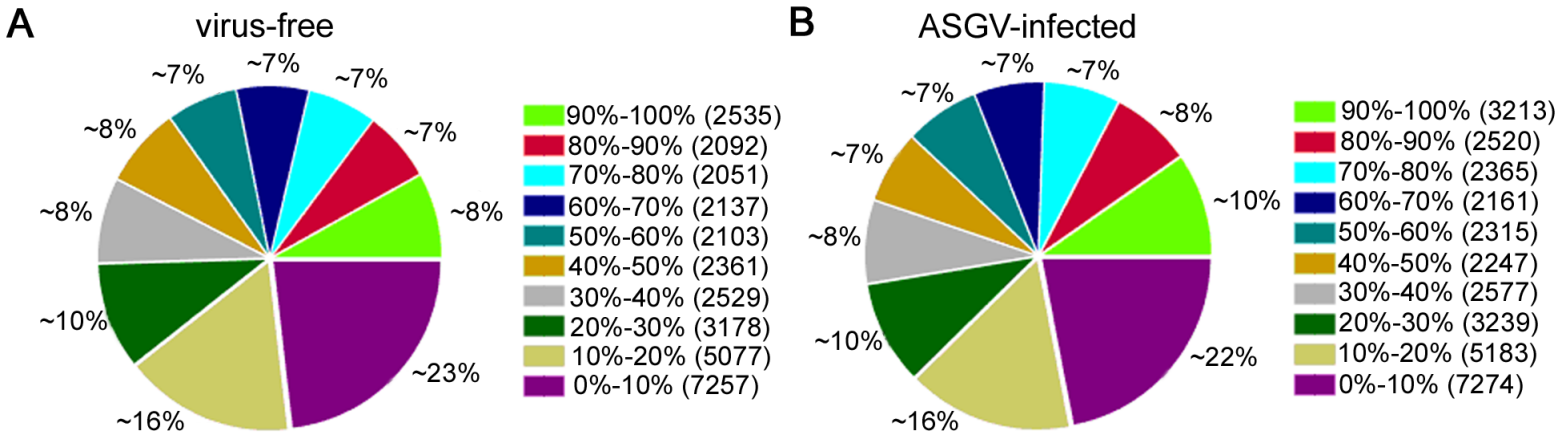
Gene coverage statistics of the two RNA-seq libraries: virus-free and ASGV-infected.

The apple *in vitro* plantlets samples were confirmed to be virus-free or only ASGV-infected by both RT-PCR and Northern blot before library construction (data not shown). RNA-seq generated 7.6 million and 7.4 million raw tags, respectively, for the virus-free and ASGV-infected apple plantlet samples (Table 1). After removal of low-quality reads (see Materials and Methods), the total numbers of tags per library ranged from 7.3 million to 7.5 million. The total clean reads mapped to the apple genome sequence [25] were 4.2 million and 5.0 million for virus-free and ASGV-infected apple plantlets, respectively. Among the mapped reads, 2.8 million and 3.3 million were unique and 1.4 million and 1.7 million were multi-positional for the two libraries, respectively. About 37.28% and 45.15% of the unique reads were mapped to a gene in the reference genome, and the numbers of mapped genes were 31,320 (54.58% of total predicted genes) and 33,094 (57.67%) for the two libraries, respectively (Table 1). The gene coverage distribution showed similar patterns for both libraries (Fig. 2), suggesting no bias in the construction and sequencing of libraries from the virus-free and ASGV-infected apple plantlets.

**Table 1:** Categorization and abundance of RNA-seq reads from libraries of virus-free and ASGV-infected apple shoots.

Summary		Virus-free	ASGV-infected
Raw reads	Total numbers	7591038	7430421
Total clean reads	Total numbers	7502805	7331064
	Total % of raw reads	98.84	98.66
Total mapped reads	Total numbers	4231368	5011174
	Total % of clean reads	56.40	68.36
Unique match reads	Total numbers	2796881	3309972
	Total % of clean reads	37.28	45.15
Multi-position match reads	Total numbers	1434487	1701202
	Total % of clean reads	19.12	23.21
Total unmapped reads	Total numbers	3271437	2319890
	Total % of clean reads	43.60	31.64
Total mapped reads	Total numbers	31320	33094
	Total % of genes	54.58	57.67

### Differentially expressed genes in response to ASGV infection

**Figure 3:**
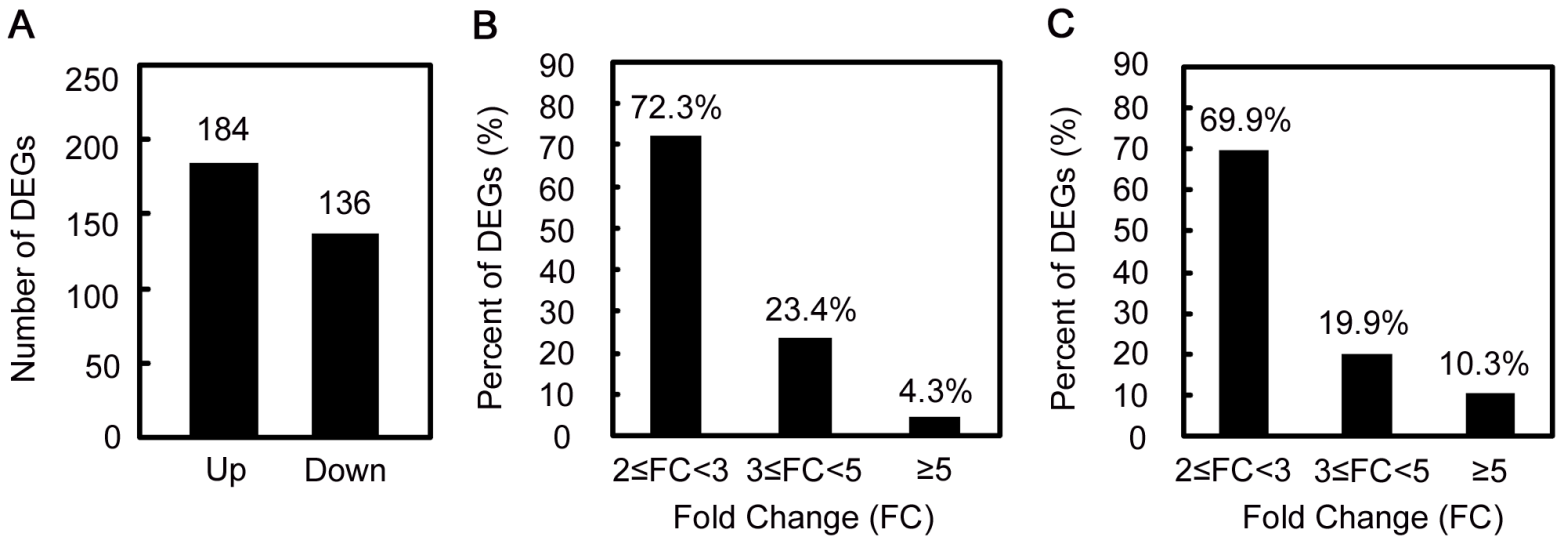
Analysis of differentially expressed genes (DEGs) response to ASGV infection. (A) Numbers of DEGs. (B) and (C) Distribution of fold changes (FC) for up-regulated and down-regulated DEGs, respectively.

To identify apple genes whose expressions changed significantly in response to ASGV infection, the differentially expressed gene (DEG) tags were analyzed. They were mapped to a total of 320 genes, with 184 up-regulated and 136 down-regulated (Fig. 3A and Supplementary Table S2). The detected fold changes (log_2_ ratio) of gene expression ranged from −13.5 to 13.7, and more than 90% of the genes were up- or down-regulated by 2–5 fold (Fig. 3B, 3C).

**Figure 4:**
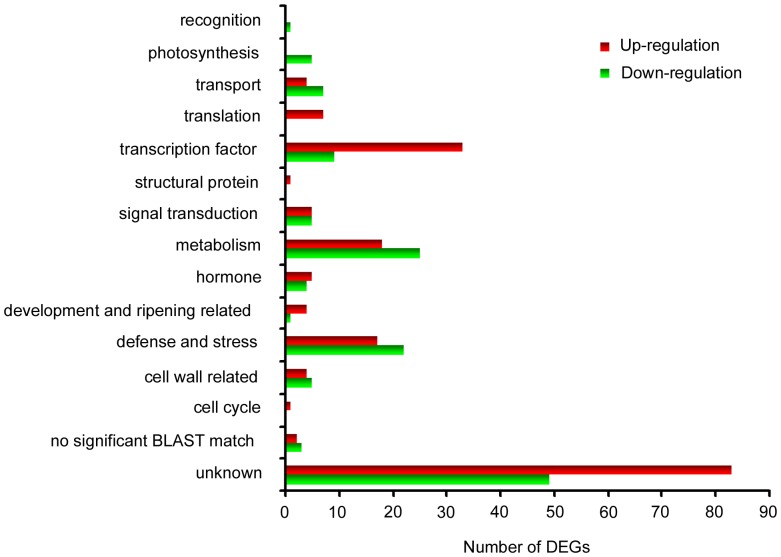
Function categorization of genes significantly induced (red) and repressed (green) in ASGV-infected apple plantlets.

The up-regulated and down-regulated genes were assigned putative functions based on the Basic Local Alignment Search Tool (BLAST) on Uniprot (Supplementary Table S2). Approximately 45% of up-regulated genes and 36% of down-regulated genes encode proteins of unknown functions. Among the genes encoding known functions, three categories showed the most prominent changes in expression – those encoding transcription factors, enzymes in primary and secondary metabolism, and defense/stress response proteins (Fig. 4). The rest of genes encode products involved in development/fruit ripening, signal transduction, transport, cell wall biogenesis, hormone metabolism/response, translation, cell cycle, structure, photosynthesis, and recognition (Fig. 4).

### Verification of differential gene expression by qRT-PCR and northern blotting

**Figure 5:**
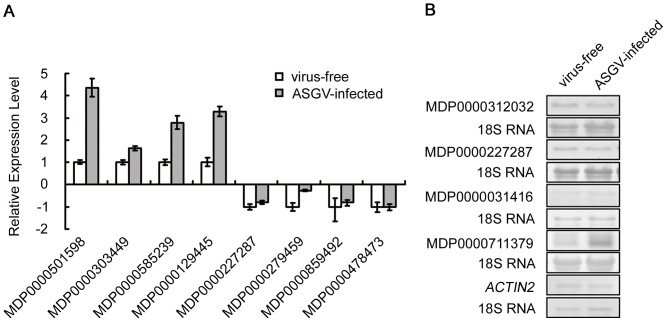
Verification of DEG data. (A) Four up-regulated and four down-regulated DEGs randomly selected were confirmed by qRT-PCR. *ACTIN2* gene was used as an internal control. Data are means and standard errors of three biological replicates. (B) Four DEGs randomly selected were confirmed by Northern blot. 18S RNA was used as a loading control.

To independently verify the differential gene expression results obtained by RNA-seq, we first used qRT-PCR to analyze the expression levels, in both virus-free and ASGV-infected apple plantlets, of 8 genes randomly chosen among the up-regulated and down-regulated ones in infected plantlets. Gene expression levels were presented as fold-changes in the infected plantlets relative to those in the virus-free samples (Fig.5A). The results showed expression pattern changes similar to those obtained from RNA-seq (Table 2) except for gene MDP0000478473. The inconsistency might be introduced by the lower sensitivity of qRT-PCR than RNA-Seq. Overall, the qRT-PCR results validated the RNA-Seq profiling results.

**Table 2:** A comparison of the results from RNA-seq and from qRT-PCR analyses of differentially expressed genes.

Gene ID	Up/Down-regulated	Fold Change (RNA-seq)	Fold Change (qRT-PCR)	Function Annotation
MDP0000501598	Up	2.07	4.35	Transcription
MDP0000303449	Up	2.19	1.63	Cell wall-related
MDP0000585239	Up	3.44	2.77	Defense
MDP0000129445	Up	4.85	3.28	Ubiquitin-related
MDP0000227287	Down	−2.00	−1.25	Secondary Metabolism
MDP0000279459	Down	−58.13	−3.70	Unknown
MDP0000859492	Down	−4.76	−1.23	Unknown
MDP0000478473	Down	−2.27	−0.99	Defense

Second, we analyzed 8 other candidate genes by Northern blotting. Among them, four genes showed hybridization signals that were consistent with their altered expression patterns in ASGV-infected plantlets based on RNA-seq (Fig. 5B and Supplementary Table S2). The other four genes exhibited no hybridization signals possibly because of their low expression levels. Overall, the qRT-PCR and northern blotting data of randomly sampled genes confirmed the RNA-seq results.

### Reduced net photosynthesis in ASGV infected apple

**Figure 6:**
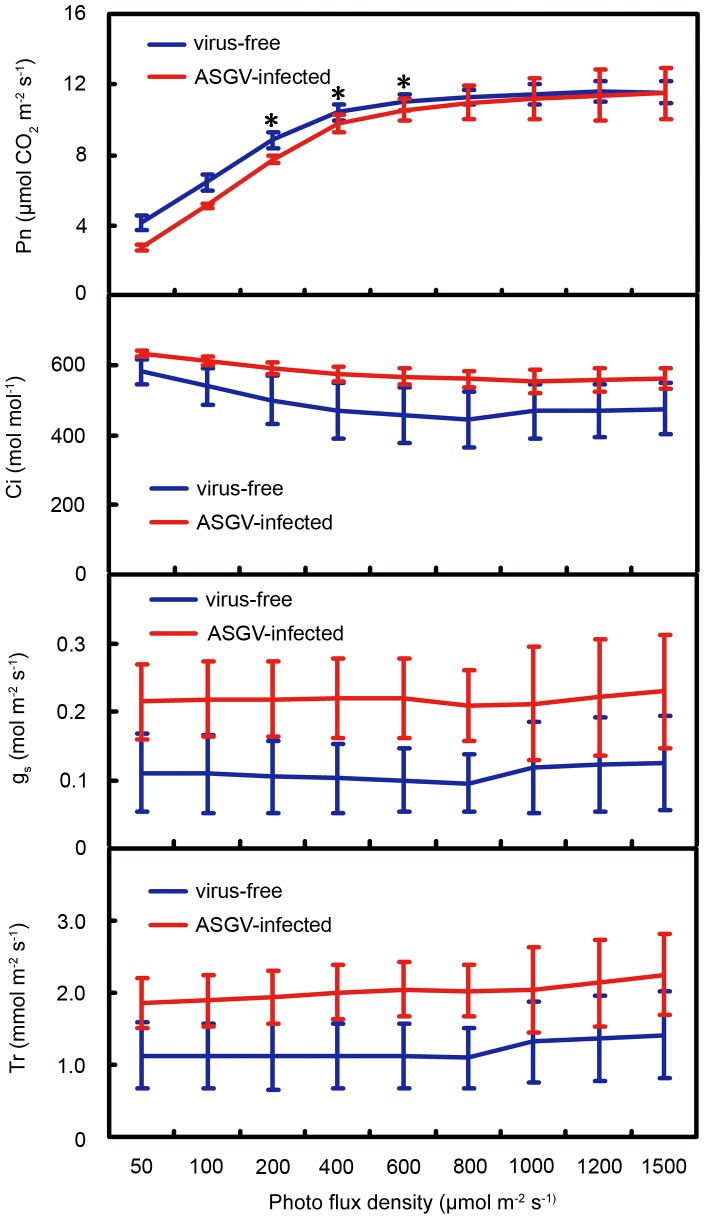
Comparison of photosynthesis between virus-free and ASGV-infected apple shoots. (A) Light response curves (net photosynthesis, Pn). (B) Intercellular CO_2_ concentration (Ci). (C) Stomatal conductance (g_s_). (D) Transpiration rate (Tr). Data represent means and standard errors based on measurements from six biological replicates. Asterisks indicate significant differences (*P*<0.05) between ASGV-infected and virus-free shoots under specific photo flux densities.

The altered expressions of selective categories of apple genes in response to ASGV infection suggest selective impacts on certain developmental, cellular and physiological processes. To test such potential impacts, we chose photosynthesis for comparative analyses between virus-free and ASGV-infected plants, given that five genes involved in photosynthesis were specifically down-regulated by ASGV infection. Rooted apple plantlets were transferred to Hoagland solution for acclimation and then planted in pots and cultured in a greenhouse. Net photosynthesis (Pn), intercellular CO_2_ concentration (Ci), stomatal conductance (gs) and transpiration rate (Tr) were measured at 14 days after plantation. The results showed that compared to virus free plantlets, ASGV infection decreased Pn by 26.1–52.7% under low light conditions (50–100 µmol CO_2_ m^−2^ s^−1^), and down-regulated Pn significantly (*P*<0.05) under 200, 400, and 600 µmol CO_2_ m^−2^ s^−1^ conditions (Fig. 6). The Ci, gs, and Tr in ASGV-infected plantlets showed some increases. However, there were no statistically significant differences in Ci, gs and Tr between virus-free and ASGV-infected plants grown under a wide range of light conditions (50–1500 µmol CO_2_ m^−2^ s^−1^) (Fig. 6). Thus, ASGV infection decreased the photosynthetic performance of apple leaves under decreasing light intensities.

## Discussion

Most apple cultivars infected by ASGV are asymptomatic, raising the intriguing question of how these hosts respond to the infection at transcriptome levels. Here we showed that ASGV infection induced extensive gene expression changes and altered some photosynthetic parameters in asymptomatic apple plantlets.

What is significant is that ASGV asymptomatic infection caused expression changes in similar categories of genes in apple as other viruses did in other plant hosts exhibiting disease symptoms [1]–[13]. These genes encode proteins involved in plant response to biotic and abiotic stress, photosynthesis, phytohormone function, metabolism, signal transduction, transcription regulation, translation, and transport. Thus, our data suggest that disease symptom development may not always simply be interpreted in terms of particular host gene expression changes. Some notable examples of genes are discussed below to further highlight this point.

Among the 320 apple genes whose expressions were altered by ASGV infection, 39 genes (12.18%) function in defense and stress responses (Fig. 4). Surprisingly, most of these genes (22/39) are down-regulated in ASGV-infected apple, in contrast to the commonly up-regulated expression of defense/stress response genes in other plant-virus interactions [2]. The down-regulated defense genes include those encoding protease inhibitor, cytochrome P450, three putative cytochrome P450s, metacaspase, Non-expressor of PR1 (NPR1) and 1, 3-β-glucanase (Supplementary Table S2). Among them, 1, 3-β-glucanase is particularly interesting because it is up-regulated in several plant-virus interaction systems [1], [6], [7], [8], [32]. The up-regulated defense genes include those encoding pathogenesis-related (PR) proteins (PR1 and PR1a) and NBS-LRR disease resistance proteins (Supplementary Table S2).

The down-regulated genes involved in stress responses include those encoding heat shock proteins (HSP83), DnaJ protein, alternative oxidase and allene oxide cyclase. Heat shock proteins are commonly up regulated by plant virus infection [2], [33]. Silencing of DnaJ and homologues appears to have opposite functions in inhibiting cell-to-cell spread of *Tobacco mosaic virus* and *Potato virus X* [34] and in enhancing susceptibility of soybean plants to *Soybean mosaic virus* [35].

Intuitively, down- or up-regulated expression of defense/stress genes is expected to lead to alterations in the dynamics of viral infection and severity of disease symptoms. However, ASGV-infected apple plants do not develop symptoms despite down-regulated expression of most defense/stress regulated genes and up-regulated expression of some of these genes. A critical question that needs to be answered in future studies is whether the opposite expression patterns of these genes lead to neutralized effects on plant growth and development to display no symptoms.

Given the critical importance of photosynthesis to plant growth and development, altered expression of photosynthetic genes is also expected to lead to disease symptoms. Many genes involved in photosynthesis are specifically down-regulated, including those encoding photosystem Q(B) protein, photosystem I P700 chlorophyll a apoprotein A1, photosystem I P700 chlorophyll a apoprotein A2, photosystem I subunit B, and ribulose bisphosphate carboxylase large chain (Supplementary Table S2). At the same time, some genes involved in the translation of proteins in chloroplast are uniquely up-regulated. These include 30S ribosomal protein S3, 50S ribosomal protein L2, 50S ribosomal protein L14, and 50S ribosomal protein L16 (Supplementary Table S2). Our experiments showed reduced photosynthesis capacity of ASGV-infected plants under low light, but not high light, conditions. Regardless of these changes, the ASGV-infected plants counter-intuitively exhibit no disease symptoms.

In summary, our transcriptome analyses reveal extensive changes in the apple gene expression patterns under ASGV infection similar to those reported for other plant-virus pathosystems [1]–[13], [36]–[39]. However, the infected apple plantlets do not develop visible symptoms, in contrast to the other plant-virus pathosystems in which the infected hosts show clear symptoms. Whether our findings may be applicable only to cultured plantlets infected by a virus or may be extended to other plant-virus interaction systems remains to be determined, but the fact that ASGV-infected mature apple trees are symptomless suggests the potentially broader implications of our findings. Thus, global host gene expression changes do not necessarily lead to viral disease symptoms. Our data suggest that the general approaches to correlate host gene expression changes under viral infection conditions to specific disease symptom, based on the interpretation of transcription profiling data and altered individual gene functions, may have limitations depending on particular experimental systems. It is possible that disease symptoms arise as a consequence of complex interactions among plant developmental stages, gene expression threshold levels as well as compensatory, synergistic and antagonistic effects of different genes.

## Supporting Information

Table S1 **Primers used in this study.**
(DOC)

Table S2 **Lists of significantly modulated genes in response to** ***Apple stem grooving virus*** **infection.**
(DOC)
