# Supplementary material for: Infection of Apple by Apple Stem Grooving Virus Leads to Extensive Alterations in Gene Expression Patterns but No Disease Symptoms
Source: PLoS One. 2014 Apr 15;9(4):e95239. doi: 10.1371/journal.pone.0095239 (PMC3988175; doi:10.1371/journal.pone.0095239)
Supplement: Table S1 — Primers used in this study. (DOC) [file pone.0095239.s001.doc]

**Table S1.** Primers used in this study

| **Virus or Gene ID** | **Forward Primer (5'-3')** | **Reverse Primer (5'-3')** | **Size (bp)** |
| --- | --- | --- | --- |
| **For detection of apple viruses and viroids** | | | |
| ASGV | GGAATTTCACACGACTCCTAACCCTCC | CCCGCTGTTGGATTTGATACACCTC | 500^a^ |
| ACLSV | GAGARTTTCAGTTTGCTMGA | AGTCTACAGGCTATTTATTATAAGT | 794^b^ |
| ASPV | ATGTCTGGAACCTCATGCTGCAA | TTGGGATCAACTTTACTAAAAAGCATAA | 370^c^ |
| ASSVd | CTCGTCGTCGACGAAGG | CAGCACCACAGGAACCTCACGG | 278^d^ |
| ADFVd | GAGGAAAACTCCGTGTGGTTC | AAGTCCACTCCCTGCCAGACC | 274^e^ |
| Actin | AACTGGGATGACATGGAGAAGAT | CCCTCCAATCCAGACACTGTAC | 797 |
| **For expression analysis by quantitative real-time PCR** | | | |
| MDP0000501598 | CGGGTCTGCTTCTTTGCTCA | AGGACTGTTTGGCGACATTGG | 180 |
| MDP0000303449 | ACCGAAGGGTAAGGGAGATGA | TGGAGGCTTGTGTGGTGGTT | 181 |
| MDP0000585239 | CAAGCAGCAGAAATAGCAACYG | AATGAGCAGCGAGCGTAGA | 222 |
| MDP0000129445 | TTTCATCACCGCCATCCGT | AGAGGCATTGTGAGAGCATTCT | 199 |
| MDP0000227287 | CAAGGGCACGAAAGTGGATT | TCCCAGTCATACACACCAAGA | 97 |
| MDP0000279459 | ATTTCTGGTCGGGTCTGTCG | CGTTATCCTTCCACTGCCRAG | 176 |
| MDP0000859492 | GCATCCAATCCCTCATCAACG | CCAGTCAGCCACACCAAAGA | 196 |
| MDP0000478473 | CTTAGGGTTTGTCTCCTTCATCAC | GACCTTTCCATCCTGTGGCTA | 152 |
| MDP0000441013 | ATTTCGGGATTGGGCACA | GCGATGTATTGGTGATGAGTAT | 256 |
| MDP0000691215 | ACAACCGAAATGGAAACCGAG | GGAACTCACTCCGAATAGTCCT | 91 |
| MDP0000597996 | ATTGGGTCTCTCTGCCAGGT | AGGGTGTCCTAAAGTTYCRCCA | 131 |
| MDP0000464827 | CCTAAAGAGATTCCGCTCCCT | GTCAGCCATAGACCTCCAGT | 176 |
| MDP0000327226 | TTCGTGAACCTGTTTCTGG | GCAACAACAATCCAAGGG | 250 |
| *actin2* | CTATGTTCCCTGGTATTGCAGACC | GCCACAACCTTGTTTTTCATGC | 82 |

^a^ **James D.** 1999. A simple and reliable protocol for the detection of apple stem grooving virus by RT-PCR and in a multiplex PCR assay. *Journal of Virological Methods* **83,** 1–9.

^b^ **Yaegashi H, Isogai M, Tajima H,** [**Sano T**](http://www.ncbi.nlm.nih.gov/pubmed?term=Sano T%5BAuthor%5D&cauthor=true&cauthor_uid=17698674)**,** [**Yoshikawa N**](http://www.ncbi.nlm.nih.gov/pubmed?term=Yoshikawa N%5BAuthor%5D&cauthor=true&cauthor_uid=17698674)**.** 2007. Combinations of two amino acids (Ala^40^ and Phe^75^ or Ser^40^ and Tyr^75^) in the coat protein of apple chlorotic leaf spot virus are crucial for infectivity. *Journal of General Virology* **88,** 2611–2618.

^c^ **Menzel W, Jelkmann W, Maiss E.** 2002. Detection of four apple viruses by multiplex RT-PCR assays with coamplification of plant mRNA as internal control. *Journal of Virological Methods* **99,** 81–92.

^d^ **Zhao Y, Niu J.** 2008. Apricot is a new host of *Apple scar skin viroid*. *Australasian Plant Disease Notes* **3,** 98–100.

^e^ **Li R, Mock R, Huang Q,** [**Abad J**](http://www.ncbi.nlm.nih.gov/pubmed?term=Abad J%5BAuthor%5D&cauthor=true&cauthor_uid=18848583)**,** [**Hartung J**](http://www.ncbi.nlm.nih.gov/pubmed?term=Hartung J%5BAuthor%5D&cauthor=true&cauthor_uid=18848583)**,** [**Kinard G**](http://www.ncbi.nlm.nih.gov/pubmed?term=Kinard G%5BAuthor%5D&cauthor=true&cauthor_uid=18848583)**.** 2008. A reliable and inexpensive method of nucleic acid extraction for the PCR-based detection of diverse plant pathogens. *Journal of Virological Methods* **154,** 48–55
