# Supplementary material for: Infection of Apple by Apple Stem Grooving Virus Leads to Extensive Alterations in Gene Expression Patterns but No Disease Symptoms
Source: PLoS One. 2014 Apr 15;9(4):e95239. doi: 10.1371/journal.pone.0095239 (PMC3988175; doi:10.1371/journal.pone.0095239)
Supplement: Table S2 — Lists of significantly modulated genes in response to Apple stem grooving virus infection. (DOC) [file pone.0095239.s002.doc]

**Table S2.** Lists of apple genes showing significantly altered expressions in response to *Apple stem grooving virus* infection. Gene ID refers to Malus×domestica Whole Genome v1.0 in Genome Database of Rosaceae (http://www.rosaceae.org/species/malus/malus_x_domestica/genome_v1.0). For each gene the best Uniprot match with the related annotation and the expression ratio value (fold change) are reported.

| **Gene ID** | **Annotation** | **log_2_(Fold change)** |
| --- | --- | --- |
| **transcription factor** | |  |
| MDP0000204889 | Bel1 homeotic protein, putative | 1.086 |
| MDP0000130173 | BRASSINAZOLE-RESISTANT 2 protein, putative | 1.205 |
| MDP0000349378 | Bromodomain protein | 2.149 |
| MDP0000704216 | AP2 domain class transcription factor | 1.883 |
| MDP0000517262 | AP2 domain class transcription factor | 1.564 |
| MDP0000273201 | AP2 domain class transcription factor | 1.508 |
| MDP0000649022 | AP2 domain class transcription factor | 1.435 |
| MDP0000143745 | AP2 domain class transcription factor | 1.330 |
| MDP0000755622 | AP2 domain class transcription factor | 1.281 |
| MDP0000939633 | AP2 domain class transcription factor | 1.020 |
| MDP0000923579 | AP2 domain class transcription factor | 2.216 |
| MDP0000315201 | C2H2L domain class transcription factor | 1.342 |
| MDP0000595671 | C2H2L domain class transcription factor | 1.757 |
| MDP0000588193 | C3HL domain class transcription factor | 1.109 |
| MDP0000501598 | C3HL domain class transcription factor | 1.052 |
| MDP0000142893 | HD domain class transcription factor | 1.083 |
| MDP0000615948 | HD domain class transcription factor | 1.342 |
| MDP0000423596 | HD domain class transcription factor | 1.045 |
| MDP0000899816 | HD domain class transcription factor | 1.935 |
| MDP0000882983 | NAC domain protein, IPR003441 | 2.204 |
| MDP0000528658 | NAC domain protein | 1.478 |
| MDP0000250597 | MYB domain class transcription factor | 1.394 |
| MDP0000159011 | MYB domain class transcription factor | 1.216 |
| MDP0000202292 | WRKY domain class transcription factor | 1.368 |
| MDP0000228304 | WRKY58 Transcription factor | 1.027 |
| MDP0000191017 | WRKY domain class transcription factor | 1.011 |
| MDP0000294489 | WRKY domain class transcription factor | 1.153 |
| MDP0000361247 | DNA-directed RNA polymerase subunit alpha | 1.141 |
| MDP0000828573 | DOF domain class transcription factor | 1.062 |
| MDP0000172441 | Zinc finger CCCH domain-containing protein 5 | 1.564 |
| MDP0000581816 | Transcription factor bHLH122 | 2.194 |
| MDP0000692406 | Transcription factor, putative | 1.158 |
| MDP0000268093 | Transcription factor, putative | 1.043 |
| MDP0000195608 | Basic helix-loop-helix protein | -2.321 |
| MDP0000314321 | Basic helix-loop-helix protein | -1.625 |
| MDP0000318013 | MYB11 | -1.131 |
| MDP0000755899 | R2R3-MYB transcription factor MYB3 | -1.419 |
| MDP0000226115 | AP2D domain class transcription factor | -1.565 |
| MDP0000133262 | DNA binding protein, putative | -1.510 |
| MDP0000309382 | NAC domain class transcription factor | -1.284 |
| MDP0000118810 | WRKY transcription factor 29-2 | -1.211 |
| MDP0000764803 | AP2/ERF domain-containing transcription factor | -1.085 |
| **defense and stress** | |  |
| MDP0000288293 | Mal d 1-like | 13.468 |
| MDP0000552270 | Cc-nbs-lrr resistance protein | 2.364 |
| MDP0000442056 | Nbs-lrr resistance protein | 1.886 |
| MDP0000585239 | NBS-LRR type disease resistance protein | 1.783 |
| MDP0000811127 | Nbs-lrr resistance protein | 1.591 |
| MDP0000920394 | Avr9/Cf-9 rapidly elicited protein | 1.369 |
| MDP0000878181 | Avr9/Cf-9 rapidly elicited protein | 1.282 |
| MDP0000711379 | Pathogenesis-related protein 1a | 1.042 |
| MDP0000031416 | BRASSINOSTEROID INSENSITIVE 1-associated receptor kinase 1, putative | 1.017 |
| MDP0000300987 | Glutathione S-transferase omega | 2.342 |
| MDP0000226223 | Glutathione S-transferase omega | 1.724 |
| MDP0000188674 | Glutathione S-transferase omega | 1.646 |
| MDP0000176510 | Glutathione S-transferase omega | 1.163 |
| MDP0000220190 | Sieve element occlusion b | 1.542 |
| MDP0000243954 | Sieve element occlusion b | 1.193 |
| MDP0000258793 | 15.4 kDa class V heat shock protein | 1.556 |
| MDP0000285927 | Auxin-repressed protein | 1.504 |
| MDP0000661381 | Cytochrome P450 | -3.923 |
| MDP0000249767 | Cytochrome P450, putative | -2.699 |
| MDP0000692178 | Cytochrome P450, putative | -1.364 |
| MDP0000478473 | Cytochrome P450, putative | -1.171 |
| MDP0000308938 | Cytosolic class II low molecular weight heat shock protein | -1.430 |
| MDP0000362505 | CII small heat shock protein 1 | -1.323 |
| MDP0000700383 | CII small heat shock protein 1 | -1.220 |
| MDP0000254260 | Heat shock protein, putative | -1.166 |
| MDP0000303430 | Heat shock protein 83 | -1.135 |
| MDP0000275331 | DnaJ | -1.087 |
| MDP0000702868 | Class IV chitinase | -1.099 |
| MDP0000471879 | Protease inhibitor | -13.720 |
| MDP0000910857 | Allene oxide synthase | -1.980 |
| MDP0000225501 | Allene oxide synthase | -1.921 |
| MDP0000399594 | 12-oxophytodienoate reductase 3 | -1.480 |
| MDP0000186231 | 12-oxophytodienoate reductase | -1.205 |
| MDP0000424398 | Allene oxide synthase | -1.229 |
| MDP0000543964 | Endo-1,3-beta-glucanase | -1.035 |
| MDP0000292425 | Non-expressor of PR1 | -1.003 |
| MDP0000150964 | Metacaspase-1 | -1.000 |
| MDP0000244591 | Alternative oxidase | -1.715 |
| MDP0000239834 | Allene oxide cyclase | -1.511 |
| **metabolism** |  |  |
| MDP0000208821 | Quinone oxidoreductase | 1.872 |
| MDP0000265131 | Quinone oxidoreductase | 1.023 |
| MDP0000515106 | Sorbitol dehydrogenase | 1.453 |
| MDP0000873573 | Sorbitol dehydrogenase | 1.001 |
| MDP0000319776 | Ubiquitin specific protease 12 | 1.279 |
| MDP0000191329 | Ubiquitin-protein ligase, putative | 1.242 |
| MDP0000239686 | Ubiquitin ligase protein cop1, putative | 1.013 |
| MDP0000704111 | U-box domain-containing protein | 1.021 |
| MDP0000184195 | UDP-glycosyltransferase 86A1 | 1.729 |
| MDP0000776779 | Alpha-xylosidase, putative | 1.737 |
| MDP0000248148 | Anthocyanidin 3-O-glucosyltransferase 5 | 2.432 |
| MDP0000229843 | Desacetoxyvindoline 4-hydroxylase, putative | 1.816 |
| MDP0000299209 | Basic 7S globulin 2 small subunit, putative | 1.476 |
| MDP0000197296 | Photoregulatory zinc-finger protein COP1 | 1.361 |
| MDP0000182000 | Flavonoid 3-hydroxylase, putative | 1.757 |
| MDP0000139525 | DNA-3-methyladenine glycosylase | 1.362 |
| MDP0000309960 | Branched-chain-amino-acid aminotransferase 2, chloroplastic | 1.156 |
| MDP0000920434 | Histone H1 | 1.108 |
| MDP0000398063 | (3S,6E)-nerolidol synthase 1 | -1.556 |
| MDP0000293842 | (3S,6E)-nerolidol synthase 1 | -3.050 |
| MDP0000474746 | Alpha/beta amyrin synthase | -1.108 |
| MDP0000227287 | Alpha/beta amyrin synthase | -1.009 |
| MDP0000140755 | Ubiquitin conjugating enzyme 2 | -1.100 |
| MDP0000265094 | Ubiquitin conjugating enzyme 2 | -1.059 |
| MDP0000127897 | Fk506 binding protein, putative | -1.183 |
| MDP0000180684 | Cytochrome P450-dependent fatty acid hydroxylase | -1.910 |
| MDP0000716496 | 4-coumarate--CoA ligase-like 5 | -1.606 |
| MDP0000025413 | Mevalonate kinase | -1.803 |
| MDP0000127630 | Chloroplast omega-3 desaturase | -1.563 |
| MDP0000156530 | Omega-3 fatty acid desaturase | -1.552 |
| MDP0000376738 | Trytophan synthase alpha subunit, putative | -1.449 |
| MDP0000133399 | Beta-fructofuranosidase, putative | -1.358 |
| MDP0000554525 | Alanine-glyoxylate aminotransferase, putative | -1.328 |
| MDP0000895663 | Anthranilate synthase, alpha subunit 1 | -1.202 |
| MDP0000672088 | Prephenate dehydratase, putative | -1.163 |
| MDP0000388769 | Phenylalanine ammonia-lyase | -1.146 |
| MDP0000312032 | 3-hydroxy-3-methylglutaryl coenzyme A reductase | -1.143 |
| MDP0000248777 | Ferrochelatase | -1.143 |
| MDP0000134397 | Arogenate/prephenate dehydratase | -1.074 |
| MDP0000273720 | Enoyl-CoA hydratase/isomerase family protein | -1.015 |
| MDP0000557646 | Rubber elongation factor | -1.003 |
| MDP0000843913 | Leucoanthocyanidin dioxygenase, putative | -1.689 |
| MDP0000162456 | Cinnamyl alcohol dehydrogenase | -1.332 |
| **development and ripening related** | |  |
| MDP0000180420 | Apple fruit acidity-related protein | 1.757 |
| MDP0000288768 | Apple fruit acidity-related protein | 1.536 |
| MDP0000691413 | Male sterility 5 family protein | 1.236 |
| MDP0000796901 | Ripening-induced protein | 1.640 |
| MDP0000236723 | Arabinogalactan protein 2 | -1.078 |
| **signal transduction** | |  |
| MDP0000298165 | Serine-threonine protein kinase, plant-type, putative | 1.736 |
| MDP0000263659 | Serine-threonine protein kinase, plant-type, putative | 1.049 |
| MDP0000148991 | Receptor serine/threonine kinase, putative | 1.542 |
| MDP0000204905 | Casein kinase, putative | 1.094 |
| MDP0000545337 | Calcium-binding protein | 1.594 |
| MDP0000580952 | Receptor protein kinase CLAVATA1, putative | -1.163 |
| MDP0000188157 | Receptor protein kinase, putative | -1.059 |
| MDP0000139683 | Diacylglycerol kinase | -1.122 |
| MDP0000187921 | Plastid jasmonates ZIM-domain protein | -1.533 |
| MDP0000780674 | Calcium binding protein | -1.482 |
| **transport** |  |  |
| MDP0000849544 | ABC transporter B family member | 1.866 |
| MDP0000309212 | ABC transporter family protein | 1.160 |
| MDP0000286751 | Mitochondrial carrier protein | 1.589 |
| MDP0000290422 | Autoinhibited H+ ATPase | 1.310 |
| MDP0000941000 | Lysine histidine transporter-like protein | -1.308 |
| MDP0000158576 | Mitochondrial phosphate carrier protein, putative | -2.213 |
| MDP0000917496 | Tonoplast intrinsic protein, putative | -1.554 |
| MDP0000261679 | Sugar transport protein 13 | -1.314 |
| MDP0000155446 | HyPRP2 | -1.131 |
| MDP0000891117 | Multidrug resistance pump, putative | -1.112 |
| MDP0000718381 | Autoinhibited calcium ATPase | -1.003 |
| **cell wall related** | |  |
| MDP0000127542 | Beta-galactosidase | 1.610 |
| MDP0000416548 | Beta-galactosidase | 1.044 |
| MDP0000738777 | Probable galacturonosyl transferase-like 10 | 1.213 |
| MDP0000303449 | Merlot proline-rich protein 2 | 1.136 |
| MDP0000614624 | Cell wall-associated hydrolase | -3.269 |
| MDP0000361180 | Cell wall-associated hydrolase | -1.773 |
| MDP0000836165 | Pectinesterase | -1.565 |
| MDP0000200773 | Pectin methylesterase 2 | -1.142 |
| MDP0000453068 | Structural constituent of cell wall, putative | -1.162 |
| **hormone metabolism and response** | |  |
| MDP0000291544 | ABA 8'-hydroxylase | 1.081 |
| MDP0000166068 | SAUR family protein | 1.091 |
| MDP0000497581 | Auxin efflux carrier component | 1.351 |
| MDP0000307853 | Cytokinin-regulated kinase 1 | 1.330 |
| MDP0000809218 | TRANSPORT INHIBITOR RESPONSE 1 protein, putative | 1.007 |
| MDP0000137705 | Gibberellin 2-oxidase | -1.463 |
| MDP0000218271 | Brassinosteroid signaling positive regulator-related protein | -1.116 |
| MDP0000228470 | Abscisic acid receptor PYL4 | -1.082 |
| MDP0000689946 | Ethylene-responsive transcription factor | -1.033 |
| **translation** |  |  |
| MDP0000361244 | 30S ribosomal protein S3, chloroplastic | 1.853 |
| MDP0000361245 | 30S ribosomal protein S3, chloroplastic | 1.539 |
| MDP0000436958 | 50S ribosomal protein L2-B | 1.533 |
| MDP0000436957 | 50S ribosomal protein L2, chloroplastic 2 | 1.476 |
| MDP0000361249 | 50S ribosomal protein L14, chloroplastic | 1.506 |
| MDP0000361241 | 50S ribosomal protein L16, chloroplastic | 1.610 |
| MDP0000291076 | Ribosomal protein L19 | 3.024 |
| **cell cycle** |  |  |
| MDP0000258414 | Cyclin-dependent kinase inhibitor 1 | 2.155 |
| **structural protein** | |  |
| MDP0000274344 | Stem 28 kDa glycoprotein, putative | 1.086 |
| **photosynthesis** | |  |
| MDP0000327226 | Photosystem Q(B) protein | -3.702 |
| MDP0000464827 | Photosystem I P700 chlorophyll a apoprotein A1 | -2.186 |
| MDP0000441013 | Photosystem I P700 chlorophyll a apoprotein A2 | -1.572 |
| MDP0000691215 | Photosystem I subunit B | -1.572 |
| MDP0000597996 | Ribulose bisphosphate carboxylase large chain | -1.458 |
| **recognition** |  |  |
| MDP0000229726 | S-locus-specific glycoprotein S6, putative | -1.410 |
| **unknown** |  |  |
| MDP0000326058 | 28 kDa heat-and acid-stable phosphoprotein, putative | 1.018 |
| MDP0000143958 | Al-induced protein | 1.553 |
| MDP0000672731 | Ankyrin repeat-containing protein | 1.148 |
| MDP0000261522 | ATP binding protein, putative | 2.130 |
| MDP0000859897 | BON1-associated protein 2 | 1.825 |
| MDP0000433094 | F-box family protein | 1.443 |
| MDP0000228529 | F-box family protein | 1.047 |
| MDP0000303736 | Guanosine-3',5'-bis(Diphosphate) 3'-pyrophosphohydrolase, putative | 1.079 |
| MDP0000808264 | Lactosylceramide 4-alpha-galactosyltransferase | 1.071 |
| MDP0000129445 | Predicted protein | 2.279 |
| MDP0000469568 | Predicted protein | 2.342 |
| MDP0000464691 | Predicted protein | 1.473 |
| MDP0000252680 | Predicted protein | 1.437 |
| MDP0000511663 | Predicted protein | 1.429 |
| MDP0000268271 | Predicted protein | 1.357 |
| MDP0000315424 | Predicted protein | 1.335 |
| MDP0000403302 | Predicted protein | 1.333 |
| MDP0000158129 | Predicted protein | 1.291 |
| MDP0000872143 | Predicted protein | 1.235 |
| MDP0000144634 | Predicted protein | 1.233 |
| MDP0000643281 | Predicted protein | 1.225 |
| MDP0000590116 | Predicted protein | 1.183 |
| MDP0000575967 | Predicted protein | 1.065 |
| MDP0000171737 | Predicted protein | 1.003 |
| MDP0000307739 | Predicted protein | 2.535 |
| MDP0000300146 | Predicted protein | 1.883 |
| MDP0000840947 | Predicted protein | 1.412 |
| MDP0000252053 | Predicted protein | 1.203 |
| MDP0000803096 | Predicted protein | 2.216 |
| MDP0000256195 | Predicted protein | 2.079 |
| MDP0000157879 | Probable protein phosphatase 2C 25 | 1.761 |
| MDP0000362327 | Putative COBL7 (COBRA-LIKE 7) | 1.307 |
| MDP0000144016 | Putative uncharacterized protein | 2.020 |
| MDP0000161388 | Putative uncharacterized protein | 1.940 |
| MDP0000767063 | Putative uncharacterized protein | 1.901 |
| MDP0000384535 | Putative uncharacterized protein | 1.827 |
| MDP0000923528 | Putative uncharacterized protein | 1.627 |
| MDP0000279037 | Putative uncharacterized protein | 1.619 |
| MDP0000148426 | Putative uncharacterized protein | 1.591 |
| MDP0000242313 | Putative uncharacterized protein | 1.583 |
| MDP0000223224 | Putative uncharacterized protein | 1.491 |
| MDP0000126335 | Putative uncharacterized protein | 1.488 |
| MDP0000156139 | Putative uncharacterized protein | 1.477 |
| MDP0000137325 | Putative uncharacterized protein | 1.453 |
| MDP0000280901 | Putative uncharacterized protein | 1.423 |
| MDP0000254055 | Putative uncharacterized protein | 1.417 |
| MDP0000208617 | Putative uncharacterized protein | 1.407 |
| MDP0000315507 | Putative uncharacterized protein | 1.356 |
| MDP0000190256 | Putative uncharacterized protein | 1.353 |
| MDP0000127306 | Putative uncharacterized protein | 1.326 |
| MDP0000819132 | Putative uncharacterized protein | 1.290 |
| MDP0000120125 | Putative uncharacterized protein | 1.281 |
| MDP0000206902 | Putative uncharacterized protein | 1.274 |
| MDP0000261375 | Putative uncharacterized protein | 1.219 |
| MDP0000296673 | Putative uncharacterized protein | 1.208 |
| MDP0000147875 | Putative uncharacterized protein | 1.198 |
| MDP0000859563 | Putative uncharacterized protein | 1.188 |
| MDP0000122634 | Putative uncharacterized protein | 1.177 |
| MDP0000377836 | Putative uncharacterized protein | 1.165 |
| MDP0000648218 | Putative uncharacterized protein | 1.164 |
| MDP0000287017 | Putative uncharacterized protein | 1.144 |
| MDP0000303602 | Putative uncharacterized protein | 1.136 |
| MDP0000514153 | Putative uncharacterized protein | 1.106 |
| MDP0000242079 | Putative uncharacterized protein | 1.105 |
| MDP0000593593 | Putative uncharacterized protein | 1.087 |
| MDP0000150279 | Putative uncharacterized protein | 1.060 |
| MDP0000304911 | Putative uncharacterized protein | 1.050 |
| MDP0000320276 | Putative uncharacterized protein | 1.040 |
| MDP0000152621 | Putative uncharacterized protein | 1.026 |
| MDP0000897855 | Putative uncharacterized protein | 1.024 |
| MDP0000451107 | Putative uncharacterized protein | 1.014 |
| MDP0000221435 | Putative uncharacterized protein | 1.011 |
| MDP0000894041 | Putative uncharacterized protein | 1.009 |
| MDP0000869399 | Putative uncharacterized protein | 1.000 |
| MDP0000289822 | Putative uncharacterized protein | 2.489 |
| MDP0000156578 | Putative uncharacterized protein | 1.424 |
| MDP0000127858 | Putative uncharacterized protein | 1.326 |
| MDP0000698017 | Putative uncharacterized protein Sb02g034560 | 1.272 |
| MDP0000316421 | Putative uncharacterized protein | 2.295 |
| MDP0000250658 | Putative uncharacterized protein | 1.738 |
| MDP0000186994 | Putative uncharacterized protein | 1.342 |
| MDP0000909888 | Ring finger protein, putative | 1.209 |
| MDP0000768772 | RING-H2 finger protein ATL2 | 1.091 |
| MDP0000361238 | no significant BLAST match | 1.471 |
| MDP0000362288 | no significant BLAST match | 1.281 |
| MDP0000324434 | 1-acylglycerol-3-phosphate O-acyltransferase ABHD5 | -1.356 |
| MDP0000263529 | ATP binding protein, putative | -1.184 |
| MDP0000174161 | DNA binding protein, putative | -3.182 |
| MDP0000485762 | EG45-like domain containing protein | -6.452 |
| MDP0000292237 | F-box protein At1g78280 | -1.050 |
| MDP0000179654 | Glutaredoxin, grx, putative | -1.041 |
| MDP0000814024 | Integral membrane single C2 domain protein | -1.463 |
| MDP0000565027 | ORF40s | -2.605 |
| MDP0000849143 | PPPDE peptidase domain-containing protein | -1.252 |
| MDP0000641053 | Predicted protein | -1.648 |
| MDP0000621569 | Predicted protein | -1.617 |
| MDP0000386613 | Predicted protein | -1.506 |
| MDP0000902434 | Predicted protein | -1.482 |
| MDP0000193401 | Predicted protein | -1.302 |
| MDP0000725999 | Predicted protein | -1.172 |
| MDP0000368719 | Predicted protein | -1.138 |
| MDP0000925349 | Predicted protein | -1.094 |
| MDP0000119199 | Predicted protein | -1.084 |
| MDP0000367087 | Predicted protein | -1.047 |
| MDP0000123418 | Predicted protein | -1.030 |
| MDP0000244335 | Predicted protein | -1.899 |
| MDP0000292084 | Predicted protein | -1.349 |
| MDP0000317152 | Putative uncharacterized protein | -1.930 |
| MDP0000279459 | Putative uncharacterized protein | -5.858 |
| MDP0000234623 | Putative uncharacterized protein | -4.330 |
| MDP0000199257 | Putative uncharacterized protein | -2.565 |
| MDP0000241358 | Putative uncharacterized protein | -1.921 |
| MDP0000835211 | Putative uncharacterized protein | -1.865 |
| MDP0000283316 | Putative uncharacterized protein | -1.864 |
| MDP0000871409 | Putative uncharacterized protein | -1.841 |
| MDP0000787216 | Putative uncharacterized protein | -1.822 |
| MDP0000176755 | Putative uncharacterized protein | -1.717 |
| MDP0000557785 | Putative uncharacterized protein | -1.644 |
| MDP0000312316 | Putative uncharacterized protein | -1.529 |
| MDP0000118943 | Putative uncharacterized protein | -1.134 |
| MDP0000856686 | Putative uncharacterized protein | -1.123 |
| MDP0000167281 | Putative uncharacterized protein | -1.091 |
| MDP0000165547 | Putative uncharacterized protein | -1.069 |
| MDP0000263680 | Putative uncharacterized protein | -1.057 |
| MDP0000859492 | Putative uncharacterized protein | -2.233 |
| MDP0000254930 | Putative uncharacterized protein | -1.670 |
| MDP0000277557 | Putative uncharacterized protein | -1.307 |
| MDP0000653408 | Putative uncharacterized protein | -1.067 |
| MDP0000248575 | Putative uncharacterized protein | -1.059 |
| MDP0000053760 | Retrotransposon protein | -1.803 |
| MDP0000936525 | Uncharacterized protein ycf68 | -1.597 |
| MDP0000499035 | Unknow protein | -2.628 |
| MDP0000188919 | no significant BLAST match | -5.413 |
| MDP0000323277 | no significant BLAST match | -1.439 |
| MDP0000352047 | no significant BLAST match | -1.006 |
| MDP0000361268 | no significant BLAST match | -1.231 |
| MDP0000653407 | no significant BLAST match | -1.193 |
